# Peer education interventions for HIV prevention and sexual health with young people in Mekong Region countries: a scoping review and conceptual framework

**DOI:** 10.1080/26410397.2022.2129374

**Published:** 2022-10-28

**Authors:** Peter A. Newman, Pakorn Akkakanjanasupar, Suchon Tepjan, Sharafdzhon Boborakhimov, Jan Willem de Lind van Wijngaarden, Nuttapon Chonwanarat

**Affiliations:** a Professor, Factor-Inwentash Faculty of Social Work, University of Toronto, Toronto, Canada; Senior Advisor, VOICES-Thailand Foundation, Chiang Mai, Thailand. *Correspondence*: p.newman@utoronto.ca; b Research Associate, VOICES-Thailand Foundation, Chiang Mai, Thailand; c Research Manager, VOICES-Thailand Foundation, Chiang Mai, Thailand; d Research Assistant, VOICES-Thailand Foundation, Chiang Mai, Thailand; Consultant, United Nations Population Fund, Berlin, Germany; e Independent Scholar/Consultant, Chonburi, Thailand; f Research Assistant, VOICES-Thailand Foundation, Chiang Mai, Thailand

**Keywords:** peer educators, adolescents, sexual and gender minorities, culture, Southeast Asia

## Abstract

WHO-recommended rights-based approaches to sexual health emphasise participatory and youth-centred processes. Among these, peer education (PE) interventions are commonly used to promote HIV prevention and sexual health for young people, particularly in low-resource, high HIV prevalence contexts. We conducted a scoping review to identify key characteristics, implementation challenges, and knowledge gaps in the literature regarding PE interventions in Mekong Region countries. Out of 6521 publications identified through database searches, 17 peer-reviewed articles were included in the review (*n* = 21,469 participants). Studies from Thailand (*n* = 7), Vietnam (*n* = 5), Myanmar (*n* = 3), Cambodia (*n* = 1), and Lao PDR (*n* = 1) included adolescent and young key populations (*n* = 11) and general population youth (*n* = 6). Findings from quantitative (descriptive) and qualitative (thematic) analysis illustrate benefits and challenges of various elements of multicomponent PE interventions in reaching vulnerable young people and improving HIV prevention and sexual health outcomes. Focal knowledge gaps emerged in regard to peer educator outcomes (increased knowledge, skill-building, empowerment); interpersonal processes between peer educators and young people (role modelling, social dynamics); and social-structural contexts (sociocultural influences, gendered power relations), which may affect PE programme implementation and effectiveness. Future research should evaluate the potential benefits of complementing evidence-based intervention approaches – focused predominantly on assessing individual-level behavioural outcomes conceptualised as external to PE programmes – with evidence-making intervention approaches that support rights-based PE programmes: incorporating a focus on dialectical and relational processes between peer educators and young people; assessing salutary outcomes among peer educators themselves; and evaluating the situated implementation of youth-engaged PE interventions in complex sociocultural systems.

## Résumé

Les approches fondées sur les droits recommandées par l’OMS mettent l’accent sur les processus participatifs et centrés sur les jeunes. Parmi ceux-ci, les interventions d’éducation par les pairs sont fréquemment utilisées pour promouvoir la prévention du VIH et la santé sexuelle des jeunes, en particulier dans les environnements à faibles ressources et à forte prévalence du VIH. Nous avons mené un examen de la portée pour identifier les principales caractéristiques, les difficultés de mise en œuvre et les lacunes de la connaissance dans la littérature relative aux interventions d’éducation par les pairs dans les pays de la région du Mékong. Sur 6521 publications identifiées par des recherches dans les bases de données, 17 articles de revues à comité de lecture ont été inclus dans l’examen (*n* = 21 469 participants). Les études réalisées en Thaïlande (*n* = 7), au Viet Nam (*n* = 5), au Myanmar (*n* = 3), au Cambodge (*n* = 1) et en République démocratique populaire lao (*n* = 1) portaient sur des populations clés d’adolescents et de jeunes (*n* = 11) et sur des jeunes de la population générale (*n* = 6). Les résultats des analyses quantitatives (descriptives) et qualitatives (thématiques) illustrent les avantages et les difficultés des différents éléments d’interventions d’éducation par les pairs à plusieurs composantes pour atteindre les jeunes vulnérables et améliorer la prévention du VIH et l’état de santé sexuelle. Des lacunes dans les connaissances centrales sont apparues concernant les effets chez les pairs éducateurs (meilleures connaissances, renforcement des compétences, autonomisation); les processus interpersonnels entre les pairs éducateurs et les jeunes (modélisation des rôles, dynamique sociale); et les contextes socio-structurels (influences socioculturelles, relations de pouvoir entre les sexes), qui peuvent influer sur la mise en œuvre et l’efficacité des programmes d’éducation par les pairs. Les futures recherches devraient évaluer les avantages potentiels d’une complémentation des approches d’intervention à base factuelle – axées principalement sur l’évaluation des résultats comportementaux au niveau individuel conceptualisés comme externes aux programmes d’éducation par les pairs – avec des méthodes d’intervention qui agissent sur les faits et soutiennent les programmes d’éducation par les pairs fondés sur les droits: en incluant une priorité aux processus dialectiques et relationnels entre les pairs éducateurs et les jeunes; en évaluant les effets bénéfiques chez les pairs éducateurs eux-mêmes; et en évaluant la mise en œuvre concrète des interventions d’éducation par les pairs avec la participation des jeunes dans des systèmes socioculturels complexes.

## Resumen

Los enfoques de salud sexual basados en derechos recomendados por la OMS hacen hincapié en procesos participativos centrados en jóvenes. Entre estos, las intervenciones de educación de pares (EP) se utilizan comúnmente para promover la prevención del VIH y la salud sexual para las personas jóvenes, en particular en contextos con escasos recursos y alta prevalencia de VIH. Realizamos una revisión de alcance para identificar las características clave, retos de ejecución y brechas de conocimiento en la literatura sobre intervenciones de EP en los países de la Región Mekong. De 6521 publicaciones identificadas en búsquedas de bases de datos, 17 artículos revisados por pares fueron incluidos en la revisión (*n* = 21,469 participantes). Estudios realizados en Tailandia (*n* = 7), Vietnam (*n* = 5), Myanmar (*n* = 3), Camboya (*n* = 1) y Lao PDR (*n* = 1) incluyeron a poblaciones clave de adolescentes y jóvenes (*n* = 11) y a la población juvenil general (*n* = 6). Los hallazgos del análisis cuantitativo (descriptivo) y del análisis cualitativo (temático) ilustran los beneficios y retos de los diversos elementos de intervenciones de EP con múltiples componentes para llegar a jóvenes vulnerables y mejorar los resultados de prevención de VIH y de salud sexual. Surgieron brechas de conocimiento focal con relación a los resultados de educadores de pares (aumento de conocimiento, desarrollo de habilidades, empoderamiento); procesos interpersonales entre educadores de pares y jóvenes (modelado de roles, dinámicas sociales); y contextos sociales-estructurales (influencias socioculturales, relaciones de poder basadas en género), que podrían afectar la ejecución y eficacia de los programas de EP. Futuras investigaciones deben evaluar los posibles beneficios de suplementar los enfoques de intervenciones basadas en evidencia -centradas principalmente en evaluar los resultados de comportamientos individuales conceptualizados como externos a los programas de EP- con enfoques de intervenciones que generen evidencia, que apoyen programas de EP basados en derechos: incorporar un enfoque en procesos dialécticos y relacionales entre educadores de pares y jóvenes; evaluar los resultados beneficiosos entre los educadores de pares; y evaluar la ejecución situada de intervenciones de EP centradas en jóvenes en sistemas socioculturales complejos.

## Introduction

Reaching young people with sexual health and HIV prevention services is crucial to achieving UNAIDS targets to end the AIDS epidemic by 2030.^1^ The Asia-Pacific region, home to nearly 60% of the world’s population,^2^ is second only to sub-Saharan Africa in the estimated number of people living with HIV (PLHIV) (∼5.8 million). Over three-quarters (78%) of new HIV infections in the Asia-Pacific region are among key populations,^3^ with young people accounting for 26% of overall incident HIV infections. Moreover, upwards of 90% of young people newly diagnosed with HIV in the region are from key populations.^4^

Greater attention and resources must be focused on HIV prevention and sexual health among young people, especially adolescent and young key populations, such as sexual and gender minorities, young people who use drugs, and young people who sell sex. Young key populations face challenges – including normative developmental concerns, legal constraints in access to services (i.e. as minors), lower levels of knowledge of HIV risks, and lesser power to mitigate those risks – that are distinct from adult key populations;^1,5,6,7^ yet they are largely left out of the research process, programme implementation, and national strategic plans for HIV prevention and sexual health.^7,8^

Sexual health is a state of physical, mental, and social wellbeing in relation to sexuality.^9^ In alignment with WHO’s^10^ recognition of sexual health as a basic human right, adolescents and youth have a right to “evidence-based comprehensive sex education” which includes human sexuality, sexual and reproductive health (SRH), and human rights and gender equality.^11^ A core principle of a rights-based approach to sexual health among young people is youth-centred pedagogy: actively engaging young people in bringing their whole selves to the education process, developing critical thinking, and engaging them as change agents in their communities.^12^ Operationalising access to sexual healthcare further requires understanding and responding to the needs of diverse young people in different sociocultural and geopolitical contexts.^9^ To these ends, peer education (PE) is a frequently used approach for promoting sexual health and HIV prevention for young people, particularly in low resource contexts.^13–15^

Peer education involves interactions between people with shared social or demographic characteristics, such as sexual or gender identity, age, or education, and behaviours such as drug use or sex work.^15,16^ While PE can be applicable in any age group, it is often applied with young people based on the principle that they can more easily reach their peers, discuss sensitive topics with them, and influence their behaviours than can older adults.^13,15^ Peer education is often seen as a low-cost approach^15^ and particularly apropos in resource-constrained settings, which are less likely to provide accessible and youth-friendly services than high-income countries.^17,13^ The potential benefits of PE are magnified for sexual and gender minority young people, and other young people living with and at risk of HIV; this is particularly the case in political and sociocultural environments in which these populations are disenfranchised and stigmatised.^6,18,19^

Several systematic reviews have assessed the effectiveness of PE interventions in promoting HIV prevention and sexual health, some focused on sexual and gender minority adults^14,20^ and adults and youth from key populations,^17^ with one focused on broader populations of young people.^15^ PE interventions have demonstrated effectiveness in improving knowledge (i.e. HIV transmission knowledge), attitudes, and intentions, with moderate effectiveness for behaviour change (i.e. increased condom use), that support HIV prevention and sexual health, and limited evidence for biological outcomes (e.g. HIV and STI incidence).^13–15,17,21^ Amid increasing evaluations of PE programme effectiveness, however, several reviews have indicated important knowledge gaps. These include lack of identification of key characteristics of effective PE interventions (e.g. standalone [PE only] vs. multi-component [PE plus structural intervention]), lack of systematic reporting of implementation challenges of PE programmes (e.g. sociocultural norms, political obstacles),^13,20–22^ and the need for further research on adapting PE interventions for diverse cultural contexts.^14,20^

Importantly, assessments of PE intervention effectiveness in increasing knowledge and behavioural risk reduction practices to promote HIV prevention and sexual health frequently overlook other salutary effects.^16,23^ For example, PE can support the empowerment of target populations of young people, and of peer educators themselves, engaging young people as change agents to effect broader shifts in sociocultural norms conducive to HIV prevention and sexual health.^13,14,20^ Additional benefits of PE can include peer educators’ increased ability to broach taboo topics about sexuality and HIV risk with others, improvements in peer-to-peer and family communications, and increased self-confidence; these may exert sustainable impacts on young people’s health beyond immediate behavioural outcomes.^15,16,22,24,25^ As fundamental aspects of a 2018 WHO-recommended, rights-based approach to sexual health for young people, it is crucial to assess the impact of PE programmes on these additional metrics.^26^ However, these outcomes are generally not included in reviews or evaluations of PE programmes, and more evidence is needed to identify the extent of their inclusion in PE programmes and to assess their impacts on overall programme effectiveness.

In the context of sustained HIV prevalence and incidence among young people in the Mekong Region, we conducted a scoping review of PE for HIV prevention and sexual health with adolescents and youth in Cambodia, Lao PDR, Myanmar, Thailand, and Vietnam. As a scoping review, we aimed to identify knowledge gaps regarding elements and outcomes of PE programmes from the perspective of a rights-based approach to sexual health, rather than to synthesise existing metrics of programme effectiveness, as well as to identify implementation challenges for PE programmes with young people in an understudied sociocultural context.

## Methods

We conducted a scoping review based on our objectives of undertaking a broad overview of the evidence^27,28^ on PE programmes in Mekong Region countries. We aimed to identify key characteristics of PE programmes and knowledge gaps^27,29^ among studies using a range of research designs and methods.^30^ Our scoping review methodology was based on the Joanna Briggs Institute (2015) manual and methods described by Arksey and O’Malley.^31^ The basic steps we undertook were the following: (1) identify the purpose of the review and the associated research question; (2) define a search strategy; (3) create *a priori* inclusion and exclusion criteria; (4) execute the search strategy; (5) chart and synthesise the data; and (6) report the results. Results are reported in accordance with PRISMA-ScR guidelines.^28^

### Research question

The following broad questions guided the scoping review: “What is the extent of the literature available on PE to promote HIV prevention or sexual health among young people in the Mekong Region countries?” and “What are key characteristics, implementation challenges, and knowledge gaps in PE programs with young people in the Mekong Region?”

### Information sources and search strategy

We developed search terms and the following list of databases to search in consultation with a specialist research librarian: Applied Social Sciences Index and Abstracts (ASSIA), Embase, ProQuest, Scopus, Sociological Abstracts, and Web of Science. Three sets of search terms were used to locate studies: one related to the population (e.g. teen*, youth), one related to the intervention (e.g. peer educat*, peer outreach), and one related to the location (e.g. Thai*, Viet*). We modified the search syntax according to the parameters of each database, including for example some databases that delimit the number of search terms. Comprehensive database searches were conducted from July to August 2019 to locate potentially relevant peer-reviewed publications, and updated in January 2021. The search was executed in English language. We also scanned reference lists of included studies to identify additional relevant sources.^28^ Table 1 shows sample search strings for ProQuest and Scopus databases.

**Table 1.** Sample search strings for ProQuest and Scopus databases

Databases	Sample search string
ProQuest	(“peer educat*” OR “peer outreach” OR “peer support*”) AND (adolescent* OR youth OR “young people” OR teen*) AND (intervention OR pilot OR program*) AND (Thai* OR Viet* OR Lao* OR Cambod* OR Myanma* OR Burm* OR “southeast asia”) NOT (India) NOT (China) NOT (Africa) NOT (“South Asia”)
Scopus	TITLE-ABS-KEY (“peer educat*”) OR TITLE-ABS-KEY (“peer outreach”) OR TITLE-ABS-KEY (“peer support*”) AND TITLE-ABS-KEY (adolescent*) OR TITLE-ABS-KEY (youth) OR TITLE-ABS-KEY (“young people”) OR TITLE-ABS-KEY (teen*) AND TITLE-ABS-KEY (intervention) OR TITLE-ABS-KEY (pilot) OR TITLE-ABS-KEY (program) AND TITLE-ABS-KEY (thai*) OR TITLE-ABS-KEY (viet*) OR TITLE-ABS-KEY (cambod*) OR TITLE-ABS-KEY (lao*) OR TITLE-ABS-KEY (myanma*) OR TITLE-ABS-KEY (burm*) OR TITLE-ABS-KEY (“southeast asia”) AND NOT TITLE-ABS-KEY (india) AND NOT TITLE-ABS-KEY (china) AND NOT TITLE-ABS-KEY (africa) AND NOT TITLE-ABS-KEY (“south asia”)

### Study selection criteria

Inclusion and exclusion criteria were developed prior to conducting the search. Included studies had to meet the following criteria: (1) focus on adolescents or young adults (under 25 years) or provide age-disaggregated data on young people; (2) include peer education, peer educators, or peer outreach; (3) conducted in a Mekong Region country (Cambodia, Lao PDR, Myanmar, Thailand, or Vietnam); and (4) focus specifically on HIV prevention or sexual health. Studies were excluded from the review if they were: (1) not published in English; (2) published before the year 2000; (3) reviews, meta-analyses, commentaries; (4) focused on adults; or (5) focused on other health (e.g. tobacco use) or non-health outcomes (e.g. family violence). We designated 1 January 2000 as a cutoff date in order to identify studies most representative of the current status of HIV and sexual health among young people. Due to the abundance of sources initially identified from China and India, we refined the search by adding these terms as exclusion criteria in the search strings.

### Study selection process

The results of peer-reviewed publications from the online database searches were uploaded into Covidence (Melbourne, Australia) software for managing reviews. Ongoing research team discussions ensured the consistent application of inclusion/exclusion criteria. Groups of two reviewers (among JDLVW, NC, SB, ST) first independently examined titles and abstracts for inclusion using the same *a priori* criteria, with discrepancies resolved by a third reviewer. Subsequently, groups of two reviewers (e.g. JDLVW/SB, ST/SB, JDLVW/ST) screened full texts of potentially eligible articles. All discrepancies between reviewers at the full-text stage were resolved by a single arbitrator (PAN).

### Data extraction and synthesis

We extracted data from the selected studies on publication characteristics (i.e. author(s), year), study setting, study population, sample size, age, study objectives, methods (i.e. qualitative, quantitative, mixed methods), PE programme description, and main study findings (see Table S1). The synthesis included quantitative (e.g. frequency) analysis of the publication year, country, focal populations, sample size, and age range; and qualitative (i.e. thematic) analysis to identify the dimensions of PE programmes reviewed. Emerging themes and subthemes were derived in an inductive process during data extraction. Two authors (PA, PAN) discussed and characterised the themes, identified themes and subthemes addressed by each study, and extracted illustrative content.

## Results

**Figure 1.**
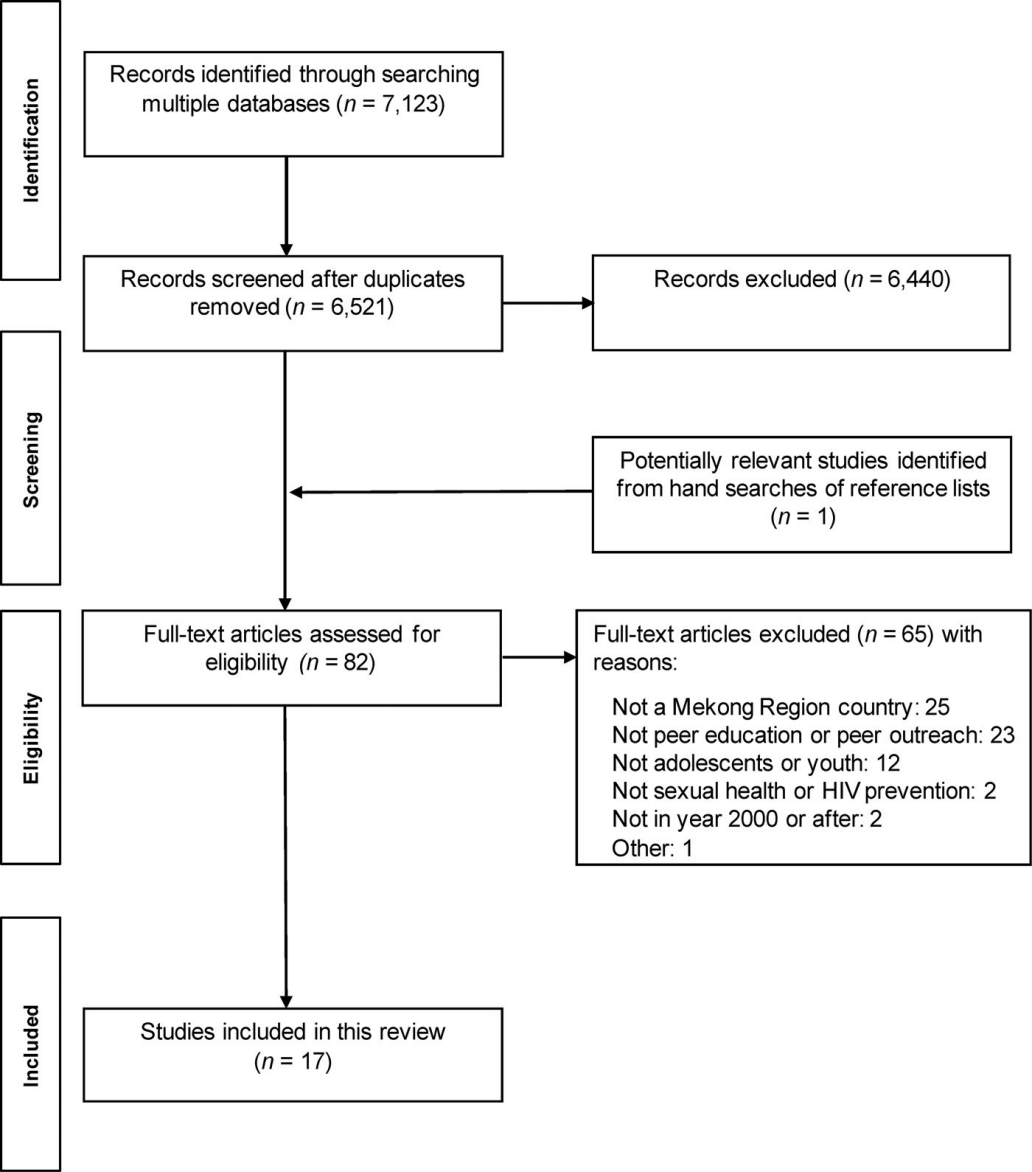
PRISMA flow diagram for scoping review of peer education for HIV prevention and sexual health with young people in Mekong Region countries

The literature search resulted in a total of 6521 citations after removal of duplicates (see Figure 1). After screening titles and abstracts, 6440 records were excluded, resulting in 81 potentially relevant full-text articles. Subsequently, 17 peer-reviewed articles were included in this review.

### Study characteristics

The majority (71%, *n* = 12) of included articles were published after 2010. Studies were conducted primarily in Thailand (41%, *n* = 7) and Vietnam (29%, *n* = 5), as well as in Myanmar (*n* = 3), Cambodia (*n* = 1), and Lao PDR (*n* = 1). The majority (53%, *n* = 9) of studies included young cisgender men and cisgender women, followed by young cisgender men only (*n* = 3), young cisgender men and transgender women (*n* = 3), and (*n* = 1 each) cisgender women only and transgender women only. Nearly two-thirds (65%, *n* = 11) of the studies were conducted with cisgender heterosexuals and six with sexual and/or gender minority youth. Overall, 65% (*n* = 11) of studies focused on key populations, the remainder (*n* = 6) on general population youth. Sample sizes of the included studies (*n* = 21,469) ranged from 100 to 5,690, with a mean of *n* = 1,279 (median = 978). Eleven studies (65%) used quantitative methods, five (29%) mixed methods, and one (6%) qualitative methods (see Table 2).

**Table 2.** Study characteristics (*N* = 17**)**

Sample size		
Range	100–5690	
Median (Mean)	978.0 (1279.4)	
** **	***n***	**%**
**Year**		
2000–2009	5	29.4
2010–2020	12	70.6
**Country**		
Thailand	7	41.2
Vietnam	5	29.4
Myanmar	3	17.6
Cambodia	1	5.9
Lao PDR	1	5.9
**Gender**		
Cisgender men	3	17.6
Cisgender women	1	5.9
Transgender women	1	5.9
Cisgender men and women	9	53.0
Cisgender men and transgender women	3	17.6
**Sexual orientation**		
Heterosexual	11	64.7
Sexual and gender minority	6	35.3
**Methods**		
Quantitative	11	64.7
Cross-sectional survey	6	
Quasi-experimental	4	
RCT	1	
Mixed methods	5	29.4
Quasi-experimental + PAR/ + focus groups/+ interviews	4	
Cross-sectional survey + focus groups	1	
Qualitative	1	5.9
Case study	1	

Note: PAR, participatory action research; RCT, randomised controlled trial.

### PE programme types and themes

The majority (88%; *n* = 15) of studies were multicomponent interventions; two were surveys of PE intervention coverage or exposure. The 15 PE interventions were community-based (*n* = 6), school-based (*n* = 3), clinic/hospital-based (*n* = 1), workplace-based (*n* = 1), and multisectoral (i.e. community- and clinic-based) (*n* = 4) (see Table S1).

Table 3 indicates overall themes and subthemes depicting dimensions of PE interventions that emerged from the studies reviewed, along with exemplar quotations. Table 4 depicts the five themes and 20 subthemes, their frequencies across articles, and which articles described benefits (+), challenges (–), or both (±) for the subthemes addressed. All 17 studies described some aspects of programme procedures and content, and programme outcome themes, with 15 (88%) addressing social-structural context. Seven studies (41%) addressed aspects of interpersonal processes and five studies (29%) peer educator outcomes, the least documented themes. Studies from Thailand, Vietnam and Cambodia endorsed all five themes, with four themes endorsed from Myanmar, and three from Lao PDR.

**Table 3.** Themes, subthemes, and exemplar quotations

Themes	Quotations [country]
**Social-structural context**	
Sociocultural influences	“ … ideas of peer identity and role modelling could not be effectively applied, because the social dynamics of youth peer groups in Cambodia were not thoroughly understood; instead, these interventions built on generic assumptions about how young people might communicate with and influence one another”^32^^, p.48^ [KH] “ … in light of the recent ‘war on drugs’ … drug users might have been reluctant to participate in research or share accurate information in such a context”^33^^, p.78^ [TH]
Stakeholder engagement	“The partnership working method is critical to success in all phases of program development … all parties need to share a common belief that they have the knowledge, capacity, and ability to work together … ”^34^^, p.69^ [TH] “ … MSM and TG women peers worked closely with health care providers and provincial health officers to conduct target mapping, plan mobile HTS [HIV testing service], and refer MSM and TG women to HIV testing services … expand[ing] access to young high-risk MSM and TG women”^35^^, p.1244^ [TH]
Gendered power relations	“Young women feel that by moving away from their families they might damage their reputation; they expressed this … in terms of their fear of being labelled as going against what our culture expects of a good woman”^32^^, p.43^ [KH] “Men tended to have better perceived access to condoms and to use condoms with regular partners more than women. This is probably due to the expectation of a stronger HIV-prevention role for men more than for women”^36^^, p.136^ [TH] “Where discussions about sexuality took place in a group dominated by young men, young women tended to stay silent … ”^32^^, p.43^ [KH]
Service linkages and access	“Exit client interviews found … almost two-fold increase in the percentage of those having their last test at a project supported clinic”^37^^, p.1^ [VN] “Some people diagnosed with HIV in our program did not register for care at the time of diagnosis, preventing us from ascertaining if they received ART and remained in care”^35^^, p.1244^
Youth-friendly services	“ … FSW [female sex worker]-oriented HIV-prevention programs focus on drop-in centers … community spaces where FSWs can relax and socialize with other FSWs and health promoters”^38^^, p.2^ [MM]
**Programme procedures and content**	
Peer recruitment	“Based on our observation at the study house, many participants enrolled in the study with or were referred to the study by their friends”^33^^, p.78^ [TH]
Peer educator training and supervision	“The group process also allowed JYLs [peer educators] to receive enough leadership skills and knowledge to initiate and implement peer-led activities for knowledge transfer … as well as to create innovative media for SRH education and HIV/AIDS prevention”^39^^, p.47^ [TH] “None of the partner NGOs had codes of conduct to advise on how peer educators should work appropriately with their peers, or procedures to deal with abuses of the PE interaction … ”^32^^, p.47^ [KH]
Peer education content and activities	“During skill-building sessions, YYLs [peer educators] were encouraged to exchange knowledge and experiences, think and solve problems critically, express their thoughts and concerns, practice communication skills, work as a team, and have open communication about sexuality in a lively learning atmosphere”^34^^, p.64^ [TH]
Access to condoms and water-based lubricant	“Free condoms and lubricants were available at clinics and drop-in centers”^40^^, p.S46^ [MM] “The services provided by peer educators were primarily distributional: delivering information either through word of mouth, pamphlets, or brochures, providing condoms … ”^41^^, p.1^ [VN]
PE programme coverage and outreach	“The limited coverage of the program may have been affected by the relatively short time frame between the baseline and the end line (6 months), leaving limited time for the intervention to take effect”^40^^, p.S51^ [MM]
**Peer educator outcomes**	
HIV and SRH knowledge and attitudes	“The result also showed higher mean scores of HIV/AIDS knowledge and attitudes towards sexual behavior … after 18 months of being youth leaders than right after training … ”^39^^, p.48^ [TH]
Skill-building	“Results from the evaluation of YLTs [peer educators] showed the positive impact of developing the confidence and personal skills for being competent YLTs [peer educators] on their own personal development”^34^^, p.69^ [TH] “Peer educators reported that they made more friends and were better able to communicate with adults. Some were able to secure fulltime employment with NGOs”^32^^, p.47^ [KH]
Empowerment processes	“I developed myself and got much knowledge and experience from taking part in the youth leader project. … I learned how to adapt myself and collaborate with others in society … Being a leader also gave me a chance to make new friends and when we worked together, the attachment between us strengthened”^39^^, p.50^ [TH] “ … the organising committee for the end-of-programme forum for peer educators, set up by the staff of RHI NGOs, was unwilling to allow young people to be members”^32^^, p.42^ [KH]
**Interpersonal processes**	
Peer educator–young people interactions	“The peer facilitators were described as ‘dynamic’, ‘friendly’, and ‘knowledgeable’”^42^^, p.1497^ [VN]
Role modelling and social dynamics	“ … if we don’t understand something during the discussion or health talk, we can ask them questions, because they are also YMSM same with us! If we ask them a question, they explained us patiently. How lovely they are!”^40^^, p.S49^ [MM] “The expectation of role modelling was not clearly articulated … ”^32^^, p.47^ [KH]
Challenges in peer engagement	“These differentials in status and power made many peer educators unapproachable, as their ability to empathize with other young people is altered and professionalized”^32^^, p.46^ [KH]
**Programme outcomes**	
Increased HIV and SRH knowledge and positive attitudes	“YYLs [peer educators] used the knowledge they had acquired from attending the capacity-building camp to give correct information to their family members about a variety of issues related to SRH”^34^^, p.68^ [TH] “More kathoy in 2006 than in 2004 could identify the role of consistent and correct condom use with regular partners in preventing STIs (97.3% vs. 89.3%, p < .001)”^43^^, p.5^ [LA]
Increased condom use	“ … prevention program components related to the workplace policy on AIDS, management of available condom services and HIV/AIDS campaigns were significantly related to increased AIDS knowledge, perceived accessibility to condoms and condom use with regular partners among young factory workers”^36^^, p.138^ [TH]
Increased access to condoms and water-based lubricant	“The services provided by peer educators were primarily distributional: delivering information either through word of mouth, pamphlets, or brochures, providing condoms, and sometimes providing clean syringes and needles”^41^^, p.1^ [VN]
Increased HIV testing	“From September 2011 through August 2016, 5,730 participants received HIV pre-test counseling; 5,629 (98%) decided to have an HIV test done, and 5,609 (99.6%) received their HIV result”^35^^, p.1241^ [TH]
Increased PrEP acceptability and uptake	“Among the 360 participants who completed the PrEP attitudes questionnaire, 235 (65.3%) reported they would definitely use PrEP if it was available; of these, 137 (58.3%) started PrEP. Of the 27 participants who said they would definitely or probably not take PrEP, 4 (14.8%) started PrEP”^44^^, p.9^ [TH]

Notes: ART, antiretroviral therapy; JYLs, junior youth leaders; MSM, men who have sex with men; NGOs, Non-governmental organisations; PE, Peer Education; PrEP, pre-exposure prophylaxis; SRH, sexual and reproductive health; STI, sexually transmitted infection; TG, transgender; YLTs, youth leader trainers; YMSM, young men who have sex with men; YYLs, younger youth leaders. Country abbreviations: KH, Cambodia; LA, Lao PDR; MM, Myanmar; TH, Thailand; VN, Vietnam.

**Table 4.** Peer education themes and subthemes by article

First author, year (country)	Social–structural context					Program procedures & content					Peer educator outcomes			Interpersonal processes			Program outcomes				N
	Sociocultural influences	Stakeholder engagement	Gendered power relations	Service linkages & access	Youth-friendly services	Peer recruitment	Peer educator training & supervision	PE content & activities	Access to condoms & water-based lubricant	PE Program coverage & outreach	HIV & SRH knowledge & attitudes	Skill-building	Empowerment processes	Peer educator–young people interactions	Role modelling & social dynamics	Challenges in peer engagement	Increased HIV & SRH knowledge & positive attitudes	Increased condom use	Increased HIV testing	Increased PrEP acceptability & uptake	Total subthemes by article
Aung, 2014 (MM)					+				+	+/-						-					4
Aung, 2017 (MM)		+		+	+	+			+	+/-				+	+		+/-	-			10
Broadhead, 2009 (VN)		+				+				+								-			4
Chamratrithirong, 2017 (TH)			+/-						+/-	+							+	+/-			5
Fongkaew, 2007 (TH)	-	+			+		+	+			+	+	+	+/-	+	-	+				12
Fongkaew, 2011 (TH)		+					+	+			+	+	+				+				7
Khoat, 2003 (VN)		+/-		+/-		+	-	-	+	+/-								+/-			8
Knibbs, 2009 (KH)	-	+	-			+	+/-	-	+	+		+/-	+/-	-	-	-					13
Longfield, 2011 (LA)			-		+				+	-							-	+			6
Mimiaga, 2016 (VN)			+	-		+	+	+		-		+		+/-	+			+/-	+/-		11
Ngo, 2013 (VN)		+		+	+	+				+							+/-	+	+		8
Oldenburg, 2014 (VN)						+			+	+/-						-				+	5
Ongwandee, 2018 (TH)			-	+		+				+										+	5
Sherman, 2009 (TH)	-	+		+	+	+		+	+	+				+				+			10
Thano, 2013 (TH)		+	-			+	+	+			+						+	-			8
Veronese, 2018 (MM)						+				+									+/-		3
Wasantioopapokakorn, 2018 (TH)		+		+/-	+					+/-									+/-		5
**Total by subtheme**	3	10	6	7	7	11	6	7	8	14	3	4	3	5	4	4	7	9	4	2	
**Total by theme**	**33**					**46**					**10**			**13**			**22**				**124**

Note: -, challenges; +, benefits; +/-, benefits and challenges; PE, peer education; PrEP, pre-exposure prophylaxis; SRH, sexual and reproductive health; Country abbreviations: KH, Cambodia; LA, Lao PDR; MM, Myanmar; TH, Thailand; VN, Vietnam.

### Social-structural context

Social-structural context denotes the array of social, cultural, and political influences, and healthcare services, that constrained or enabled PE programmes, comprising 27% (33/124) of the content among five themes.

#### Sociocultural influences

Sociocultural influences, included in three of 17 articles (18%), comprised social, political, and cultural influences that served as enablers or barriers to PE. These subsumed adult control of PE programmes, adults’ lack of understanding of young people, and stigma and criminalisation of certain risk behaviours. Social and political constraints, often expressed in adult control over ostensibly peer-level interventions, limited the recruitment and selection of peer educators, types and content of PE programme training, and peers’ involvement in programme leadership and decision-making. As a study in Cambodia revealed, “the peer education … delivered was constrained by the social and political context … and the pervasive adult control over their interventions,” with “the social dynamics of youth peer groups in Cambodia … not thoroughly understood”.^32^^, p.49^

Sociopolitical influences were also evident in some young people’s reluctance to participate in PE programmes, and constraints on disclosure of stigmatised and criminalised behaviours. For example, Thailand’s previous “war on drugs” was described as resulting in lower participation among young people who use drugs.^33^ Peer educators also experienced stigma – “slander and gossip, with accusations by adult members of the community that they knew too much about sex and/or were infected with HIV”^32^^, p.47^ – as a result of their involvement with HIV and sexual health programmes.

#### Stakeholder engagement

Ten of 17 studies (59%) addressed stakeholder engagement, indicating that mutual involvement, collaboration, and partnership with various community stakeholders fostered peer educator success, as well as supporting programme sustainability and policy change. Four studies described engagement at the community level with local community leaders, health authorities, service providers, and/or police, indicating this supported buy-in and resource mobilisation critical to programme implementation. For example, in a PE intervention for young methamphetamine users, “the research took place in an unmarked building and the Thai research team worked closely with the police to ensure participant safety”.^33^^, p.78^ Alternately, some programmes were rendered unstable due to lack of stakeholder support: “… not all of the provincial-level stakeholders (e.g. local leaders and law enforcers) had fully accepted and supported the PE programs … [making] it difficult to obtain funds”.^41^^, p.12^

Two studies, both in Thailand, described partnerships with schoolteachers, administrators, and parents, that promoted peer educator competencies and programme success by creating an enabling environment, such as “changing school policy and mobilizing resources necessary for successful SRH education and HIV prevention among early adolescents”;^39, p.53^ teachers’ direct “support, assistance, and consultation” of peer educators; and involving parents in PE programme development thereby “reducing the conflicts and obstacles in teaching sex education to early adolescents”.^34, p.68–69^ Notably, these stakeholder engagement processes promoted the sustainability of HIV prevention activities in schools.^39^

Three studies described stakeholder engagement with healthcare providers and clinics which yielded multilevel benefits. This included peer educators being “ … trained by medical professionals in HIV, hepatitis and sexually transmitted diseases”,^45, p.835^ training staff in working with key populations, and supporting clinic policies that promote confidentiality and informed decision-making by young people.^40^ Healthcare provider engagement also served to expand the reach and competencies of traditional healthcare services to support youth-friendly services.

#### Gendered power relations

Six of 17 studies (35%) addressed the impact of gendered power relations on PE group dynamics and young people’s involvement. Gender norms emerged as constraints on HIV prevention and SRH behavioural outcomes, above and beyond individual knowledge and attitudes, such as, “women’s possession and initiation of use of condoms may be more stigmatized”.^36, p.136^ Sociocultural gender norms created tensions around PE programme involvement, particularly for young women who “might damage their reputation” in their community; however, young men were also rendered vulnerable by gender norms that enable risk-taking behaviour, “in a culture in which it is seen as ‘macho’ to take risks, and to have multiple sexual partners”.^32^^, p.43^ Nevertheless, PE interventions often seemed not to recognise or address sociocultural gender norms, nor to broach this topic with peer educators. For example, “most project staff were unable to describe how the SRH needs of young men and of young women might differ, and how interventions should address these differences”.^32, p.43^ Cultural gender norms impact not only young people, but may be evinced in the apparent lack of awareness or knowledge about gendered differences among programme staff and peer educators, and the lack of attention to gender in the content and methods of interventions focused on young people’s sexual health.

While several studies included both young cisgender men and women, and cisgender men who have sex with men (MSM) and transgender women, only one study provided gender-disaggregated results. A multicomponent intervention with young factory workers in Thailand revealed young men had fourfold higher odds of perceiving access to condoms, and nearly twofold higher odds of using condoms with regular partners than women;^36^ however, there was no elaboration on implications for PE interventions.

Finally, the pairing of young MSM (YMSM) peer educators with YMSM peers in Vietnam exerted a positive influence, as “MSM in the study felt they could relate to the other group members and were comfortable discussing sensitive topics”.^42, p.1497^ However, transgender women in Lao PDR, while indicating a preference for a cisgender woman or trans woman interviewer, “may have actually felt less comfortable providing sensitive information to a peer (*kathoy*)”.^43, p.10^ This suggests the complexities of gender relations, possible cultural differences, and the need to expand understanding of gendered power relations to sexual and gender minority populations.

#### Service linkages and access

In seven of 17 studies (41%), positive impacts of integrating PE programmes with community-based HIV, STI, and/or SRH services (*n* = 3 studies), hospital-based clinics (*n* = 2), and “one-stop shops” within community or hospital settings (*n* = 2) were identified in increased programme access and uptake, as well as challenges. In Thailand, peer educator-referred participants versus self-referrals/walk-ins at HIV testing sites were significantly younger, more likely to use mobile (vs. hospital) testing sites, and more likely to be testing for the first time, with the highest HIV incidence among those less than 25 years old.^35^ This suggests that in addition to increasing uptake of HIV testing, PE interventions may expand access to YMSM and young transgender people at risk. In a mixed methods study of a PE intervention among YMSM in Myanmar, qualitative findings corroborated the benefits of peer referrals suggested by positive trends of increases in HIV testing uptake: “If we are interested in blood [HIV] testing, peer educators accompanied us to go to the clinic; that’s the point I like most … feeling like we are not alone … ”.^40, p.549^ Alternately, YMSM in Vietnam described the separation of HIV testing and PE programme sites as a barrier to uptake.^42^

#### Youth- and key population-friendly services

In seven of 17 studies (41%), the integration of PE programmes with health services, often through drop-in centres, supported youth-friendly services and promoted uptake of HIV and STI testing and care for young gay men, young transgender people, and sex workers. A PE programme in Lao PDR included development of “drop-in centers as safe spaces where *kathoy* [transgender women]-specific health information and referrals to *kathoy*-friendly health services could be obtained … ”.^43, p.3^ A YMSM in Myanmar reported: “ … the drop-in center is great space, near to the clinic, friendly, free and wonderful place … . We can stay, rest, play games, listen [to] music” and “can go to the clinic easily”.^40^^, p.S49^

### Programme procedures and content

This theme comprises the functioning of PE programmes in terms of recruitment, training, coverage, and activities, addressed in 31% (38/124) of the overall thematic content.

#### Peer recruitment

Nearly two-thirds (11/17; 65%) of studies indicated the effectiveness of peer educators in reaching other young people, particularly “vulnerable hidden populations … not reached by mainstream HIV prevention messages”.^46, p.110^ Recruitment methods included chain-referral “from local organizations working with YMSM” in Myanmar^40, p.S47^ and venue-based sampling in Vietnam.^41^ Involvement of young people living with HIV promoted recruitment and exerted a positive influence on young people: “ … people who speak openly about their HIV-status can be very persuasive promoters for HIV prevention”.^41, p.13^ The few studies describing peer educators’ role in retention indicated high rates of study completion, follow-up, and positive evaluations from young people.^33,42^

#### Peer educator training and supervision

Over one-third (6/17; 35%) of the included studies described peer training as a multifaceted process comprising the acquisition of new knowledge, skill-building, understanding ethical guidelines, building confidence, and developing group norms. In Vietnam, “ … a week-long in-depth training” included “content and delivery of the intervention … group-facilitation skills and ethical research conduct”,^42, p.1495^ while in Cambodia, “Peer educators gained skills and knowledge, small financial incentives … ”.^32, p.47^ Two studies described challenges resulting from a lack of explicit training, guidelines, and supervisor expectations about peers’ behaviour and roles,^41^ such as relying on peer educators to share HIV test results with young people – an “unfair psychological burden”.^32^^, p.48^

#### Peer education content and activities

Seven of 17 studies (41%) addressed PE programme content and activities, with five describing successful involvement of young people and adult stakeholders, including early adolescents, teachers, and parents. This included formative research, such as “key informant interviews with the target population as well as individuals who worked with youth in such arenas as criminal justice, youth focused organizations, and drug treatment programs,” pilot testing, and “focus groups … to garner feedback”.^33, p.72^ PE training sessions characterised as effective encompassed interactive group activities, skill-building, problem solving, and team-building, including “communication skills … practiced in roleplays”^33, p.72^ and a “group format” endorsed by peer educators “because they enjoyed listening to and sharing stories … ”.^42, p.1497^ Two studies identified challenges, with more didactic approaches that lacked skill-building elements deemed less effective in training and programme implementation: “ … although some teams distributed condoms, few provided demonstrations on correct condom use, and few provided skills on negotiating condom use with sex partners”.^41^^, p.14^

#### Access to condoms and water-based lubricant

Nearly half (8/17; 47%) of studies indicated that multicomponent PE interventions provided greater access to condoms and water-based lubricant. This was evident from young people’s self-reports – among transgender women in Lao PDR, “perceptions of [condom] availability increased significantly … ”^43, p.5^ – and programme data, particularly in interventions that included community-based organisation (CBO) drop-in centre and clinic sites. In a multicomponent PE intervention with YMSM in Myanmar, a larger proportion of participants in intervention sites (66%; *n* = 55/84) received condoms and lubricant from peer educators than were received among those in control sites (19%; *n* = 8/42).^40^

#### PE programme coverage and outreach

Most studies (14/17; 82%) addressed programme coverage, which was impacted by the geographical disbursement and population density of locations, the numbers of vulnerable young people targeted for outreach or recruitment, the transitory nature of certain populations (e.g. sex workers),^38^ along with formative research and the time and resources allotted for peer educators to conduct recruitment. Multisectoral and youth-friendly programmes facilitated coverage of vulnerable populations, such as in Myanmar, where YMSM were linked with community- and clinic-based services that were youth-friendly and tailored to meet their specific needs.^40^ In Vietnam, formative research engaged local stakeholders, including youth, to develop “a culturally appropriate VCT service model” and “integrated services … tailored to meet client specific needs”.^37, p.11^

Five studies described HIV and sexual stigma as barriers to engaging young people in PE programmes for HIV prevention and sexual health, with some YMSM, as in Myanmar, being “afraid of being seen by others … ”.^40, p.S50^ Similarly, in Vietnam, some MSM who “identify as straight were more susceptible to stigma and therefore less likely to be willing to engage in peer education services in public settings”.^46, p.110^

### Peer educator outcomes

This theme comprises PE programme impacts on peer educators’ knowledge, attitudes, skills, and empowerment, representing 8% (10/124) of the overall thematic content.

#### HIV and SRH knowledge and attitudes

Three of 17 studies (18%) described peer educators’ gaining knowledge and developing more positive attitudes toward HIV prevention and sexual health as a function of their training. These programmes combined didactic and experiential elements, such as a sexuality class, skill-building exercises, and meeting with PLHAs: “accurate perception of the risk of HIV/STIs infection and its severity was enhanced by interviewing HIV/AIDS patients at a hospice temple”.^48, p.59^ Two studies reported peer educators’ enhanced knowledge and attitudes through their involvement in developing and implementing the PE intervention, in addition to their training.^34,39^

#### Skill-building

In four of 17 studies (24%), peer educators gained interpersonal skills in communication, including comfort and confidence in talking about sensitive topics around sex and HIV; one study also described related challenges. Peer educators acquired skills, such as creating digital media (“edutainment”) “to make the content more interesting and attract others”.^39, p.50^ and ability to “communicate with adults”, which also served as a platform to expand their social networks and find fulltime employment.^32, p.47^ Some peer educators specifically demonstrated “the confidence to provide answers when their friends asked questions about sex”.^34, p.46^ However, limitations emerged alongside strengths in a Cambodian study: “ … peer educators were most comfortable in explaining factual information but were less confident talking about sexual relationships and puberty”.^32, p.46^

#### Empowerment processes

Three of 17 studies (18%) identified empowerment processes as among the indicators of PE programme success. Evidence emerged in peer educators’ increased confidence and leadership capacities, critical thinking, problem-solving, and collaboration. For example, 16–18-year-old peer educators described “taking responsibility for work” and improving “analytical thinking, planning, coordinating, punctuality … ”.^39, p.49^ In Cambodia, confidence building was evidenced in “being able to speak in public” or reporting ability “to negotiate with a sexual partner,” which were seen as indicators of program success.^32, p.42^ In one Thai study, peer educator empowerment was described vis a vis the family: “families trusted them more than before, had more respect for their rights, and listened to them more when they shared their problems … ”.^34, p.68^ Alongside positive elements documented in a Cambodian study, constraints to empowerment were described in limits to young people setting PE programme agendas and “peer educators’ tendency to decontextualize the issues they talked about … [as] they had not had the opportunity to resolve their own concerns about sexual issues”.^32, p.42,47^

### Interpersonal processes

Interpersonal processes comprised interactions and social dynamics between peer educators and young people, represented by 10% (13/124) of overall thematic content.

#### Peer educator–young people interactions

Five of 17 studies (29%) described positive (2 studies), negative (1 study) and mixed (2 studies) elements in the interactions between peer educators and young people. Sexual and gender minority young people, in particular, recounted positive experiences with peer educators who shared their identities, which facilitated their comfort and openness: “All but 6 [of 100] men [YMSM] only felt comfortable discussing personal experiences or sensitive topics with the peer-facilitators”.^42, p.1497^ In Myanmar, “YMSM participants … were happy with the health education that they [peer educators] provided and felt that they were an approachable resource”.^40, p.S48^

However, interactions between young people and peer educators were not universally positive and health promoting: “ … not necessarily the positive and supportive exchanges assumed in the simplistic view of social dynamics between young people that underlies peer-education practice”.^32, p.45^ For example, some peers reported tensions in maintaining their own friendships: “ … they felt that they had to work harder than their peers, resulting in less time to go out with their friends”.^34, p.65^ One study reported a “hierarchical educational model” through which “new knowledge gave peer educators status and power,” making them less approachable.^32, p.46^

#### Role modelling and social dynamics

Of the four of 17 studies (24%) addressing this subtheme, three described positive and one, negative impacts. Positive peer educator–young people interactions were characterised by collaborative problem solving and role modelling. In Vietnam, YMSM participants and facilitators discussed sexual “triggers”, strategies for reducing sexual risk behaviours, and collaborated in developing plans for implementation, with researchers paying specific attention to “rapport building”, engagement of participants in the same group throughout the intervention, and hiring, training, and support of experienced peer facilitators from the MSM community.^42^ In a school-based PE intervention in Thailand, “positive feedback and assessment from [10–14-year-old] Younger Youth Leaders [YYLs]” made 16–20-year-old Youth Leader Trainers “proud of taking such facilitating roles” and the latter “were pleased to see changes among the YYLs and thought that they would be able to serve as good role models … ”.^34, p.65^ Congruent with the participatory action research model adopted by this project, the intervention focused as much on cultivating salutary peer educator–young people interactions (intervention processes) as on changes in knowledge and attitudes (intervention outcomes), with both process and outcome evaluations planned and implemented.

Alternately, low effectiveness of a PE programme in Cambodia was attributed to failure to consider existing social dynamics and cultural context. In contrast to participatory processes and collaboration, a public discourse rife with moralising responses to drug use and HIV sexual risk in a context characterised by hierarchy and authoritarianism rendered adult programme staff’s approach and perspectives incompatible with “externally driven” non-governmental organisations’ “rhetoric of empowerment”.^32, p.41^ “Peer education was taken on … as if it were a proven and transferable method for health promotion, rather than a social process that needed to be rooted in the specific social dynamics of diverse peer groups”.^32, p.48^ Rather than declaring fundamental incompatibility between PE programmes and the Cambodian context, the authors suggest better attuning PE programmes to existing social dynamics among young target populations and with adults, and providing ongoing support to enable adults and programme leaders to “relinquish control” over PE programme implementation.^32, p.49^ This can be understood as a shift from an evidence-based to an evidence-making intervention approach, given the change in focus from the implementation and fidelity of a prescribed programme and outcomes to sociocultural processes of programme development and implementation in a particular context and timeframe.

#### Challenges in peer engagement

Four of 17 studies (24%) described difficulties in recruiting and engaging young people from marginalised populations. For example, younger male sex workers in Vietnam were described as “a particularly vulnerable group, and future work should consider how to engage them in HIV prevention services”.^46, p.110^ Lower than expected participation by female sex workers in Myanmar was reported, as “attending peer educator talks ranged from 15 to 50%”.^38, p.1^ Nevertheless, while some studies enlisted support from schools and other community stakeholders to facilitate participation by young people, peer educators sometimes bore the onus of programmematic failures: “ … where it was difficult to get young people to engage, this was perceived as a failure on the young people’s part, rather than a failure of the project to make itself relevant to them”.^32, p.42^

### Programme outcomes

Programme outcomes comprised impacts on young people’s knowledge, attitudes, and behaviours that promote HIV prevention and sexual health, 24% (30/124) of content across themes.

#### Increased HIV and sexual reproductive health knowledge and positive attitudes

Of the seven studies (41%) addressing HIV and SRH knowledge and attitudes, four described positive, two both positive and negative, and one only negative outcomes. Positive outcomes were evidenced in a university-based one-session peer educator-delivered intervention in Thailand, which showed significant increases in HIV prevention knowledge and attitudes at post-test and two-month follow-up.^48^ A multicomponent school-based PE intervention in Thailand similarly indicated significant increases in HIV knowledge and attitudes towards sexual behaviour.^39^ A two-year community- and factory-based integrated PE intervention project in Vietnam revealed significant increases in HIV prevention knowledge items (e.g. effectiveness of consistent condom use) and positive attitudes toward HIV testing, at 24 months; however, some knowledge items revealed significant decreases (e.g. HIV transmission by anal sex).^37^ Overall, these studies reveal positive impacts on knowledge, and also demonstrate the importance of sustaining and evaluating interventions over time to support retention of positive outcomes among young people.

#### Increased condom use

Over half (9/17; 53%) of the studies addressed condom use outcomes, with five indicating increases and four no evidence of change. A multicomponent PE intervention with community- and factory-recruited youth in Vietnam found a significant increase in condom use intentions.^37^ Similarly, a multicomponent PE intervention with young transgender women in Laos showed a significant increase in those who reported condom use for anal sex with boyfriends and with casual partners;^43^ and a PE intervention with MSM in Vietnam reported a statistically significant reduction in the number of condomless sex acts from baseline to three-month follow-up.^42^ In a PE programme with YMSM in Myanmar, amid some evidence of changes in knowledge and attitudes, no significant effects were demonstrated on condom use in intervention vs. control townships; the authors noted constraints due to the 6-month study time frame and the existence of other concurrent HIV interventions in the townships.^40^ Notably, only one PE intervention assessed gender differences in outcomes: among young factory workers in Thailand, men were nearly twice as likely as women to use condoms with regular partners,^36^ although implications for prevention were not addressed.

#### Increased HIV testing

Four of 17 PE programmes (24%) assessed HIV testing outcomes, all of which indicated increased testing uptake, intentions, or acceptability; however, three studies also identified challenges to promoting HIV testing. A multicomponent PE intervention with young people in Vietnam indicated significant increases in HIV testing intentions and HIV testing uptake.^37^ In addition to overall increases in HIV testing in a multicomponent PE intervention with YMSM and young transgender women in Thailand, those testing in project-sponsored mobile clinics were significantly younger and more likely to be tested for the first time than those tested in hospitals.^35^ A study in Myanmar revealed opportunities as well as challenges for HIV testing among YMSM and young transgender women, with a high level of acceptability (86%) indicated for peer-delivered HIV testing among those previously untested, but 50% lower acceptability among those with more than five casual partners in the past three months.^47^ Moreover, in Thailand, fragmentation between HIV testing and treatment services was identified as a barrier to treatment initiation,^35^ which may disincentivise testing.

#### Increased PrEP acceptability and uptake

Two studies (12%) assessed PrEP acceptability or uptake. A survey of male sex workers in Vietnam identified a significant association between previous contact with a peer educator and willingness to use PrEP.^46^ In a multicomponent PE intervention with MSM and transgender women in Thailand, a majority of participants (69% of 534) who tested HIV-negative agreed to learn more about PrEP, with 46% (*n* = 167) of these initiating PrEP.^44^

## Discussion

This is the first scoping review of PE for HIV prevention and sexual health among young people in the Mekong Region, despite the substantial and ongoing HIV prevalence and incidence among young key populations. Our findings suggest benefits as well as challenges for multicomponent PE interventions (88% of the studies reviewed) in reaching young key populations and effecting positive changes in knowledge, attitudes, behavioural intentions, and access to services that support HIV prevention and sexual health, with mixed evidence on effecting behaviour change. The review also reveals insights into several important but largely underexplored dimensions of PE programmes and implementation challenges, both in the Mekong Region and in the broader international PE literature: PE programme impacts among peer educators, interpersonal/relational processes between peer educators and young people, and sociocultural influences.

Our findings on the influences of PE programmes on peer educators address a substantial gap in the PE literature.^15,49^ While few of the reviewed studies described outcomes among peer educators themselves, those studies that did identified them as critical to PE programmes. To the extent positive outcomes are achieved only among peer educators, rather than the much broader target populations of young people, it may be argued that PE interventions are not a cost-effective approach. However, the benefits of PE interventions may be understood to encompass outcomes beyond those that are typically the focus of time-limited research investigations, that is, immediate to short-term knowledge, attitudes, and behaviour change among target populations of young people.^50^ Consideration and assessment of benefits to peer educators is aligned with a rights-based approach to young people’s sexual health; this places distinct value on peer educator training, skill-building, and participation in programme planning and implementation that supports young people as change agents.^12^ Moreover, these salutary peer educator outcomes may yield benefits that complement, and in some cases may be more sustainable than, knowledge and behaviour change outcomes among target populations. Similar to our findings, a systematic review and meta-analysis of peer interventions for youth sexual health education in 10 high-income countries across 4 continents (*n* = 18,389) revealed scant attention to outcomes among peer educators (2 of 15 studies) and suggested the importance of assessing effects on peer educators and their potential impacts on sustaining PE programme outcomes in communities.^51^

Cultivation of peer educator skills in health communication, collaboration, and leadership, and group socialisation into positive norms about sexuality and gender equity among peer educators, can exert beneficial effects on their own health.^16,50^ A few included studies further documented positive impacts among peer educators that extended to their friends, families, and communities, although this was outside the scope of most outcome assessments. Similarly, several studies identified benefits at the community level in peer educators’ contributing to the introduction and expansion of youth-friendly HIV and sexual health services in schools, clinics, and CBOs. These peer group, familial, and community impacts can promote shifts in social norms, which reduce stigma and enable conversations about HIV and sexual health with peers and families, and pierce social isolation of sexual and gender minority adolescents, all of which may contribute to rights-based approaches that aim to sustain SRH and HIV prevention among young people through transformation of social-structural contexts. The assessment and evaluation of these broader impacts of PE programmes on peer educators, and their potential contribution to programme effectiveness and sustainability of outcomes, is an important direction for future research.

Another gap we identified, in exploring the interpersonal processes that transpire between peer educators and target populations, has similarly been reported in a previous review of the impact of PE and psychosocial interventions more broadly on condom use among young people in low- and middle-income countries (LMICs).^25^ In an earlier systematic review of PE programmes for adolescent sexual health education, largely focused on the USA, among 13 studies none assessed interpersonal processes between peer educators and young people.^52^ Interpersonal and relational processes may be particularly salient for PE interventions with young people, who face normative tasks around identity development and navigating risk, and sensitivities to peer group norms and expectations – more so for young key populations at elevated risk for HIV. The perceived approachability of peer educators by young people, particularly marginalised populations such as sexual and gender minorities, male and female sex workers, and younger adolescents in restrictive sociocultural and geopolitical milieus, emerged as a critical dimension of successful recruitment and programme implementation. Interpersonal rapport also facilitated role modelling and collaborative problem solving that formed the basis of successful PE interactions amid broader sociocultural constraints.

Another important dimension addressed in most of the studies reviewed was aspects of the social-structural context in which PE interventions took place. Engagement of local stakeholders (i.e. teachers, police, healthcare providers, local politicians), and linkage of PE programmes and peer educators with health and social services, some of which were developed as part of the PE intervention, supported effectiveness in what were largely multicomponent programmes. Rather than relying on PE in isolation, providing and facilitating access to competent and youth-friendly services is critical to programme effectiveness with young people.^13^ Nevertheless, few studies addressed the impact of sociocultural norms, beliefs, and practices on PE programme implementation or content, a gap similarly identified in reviews of PE for HIV prevention with sexual and gender minority adults in southeast Asia^14,53^ and globally.^20^ Notably, those studies in the present review that did address local sociocultural contexts identified their powerful influences, both as barriers and enablers of PE intervention effectiveness. Some social and cultural norms, and hierarchical models employed by some PE interventions, constrained interactions between peer educators and young people.

Sociocultural context may be particularly relevant in developing or adapting PE programmes for HIV and sexual health in the Mekong Region and other Asian countries, particularly among young people. For example, gendered power relations impact young people across cultures, including the Global North, often disadvantaging young women, young gay men, and young transgender people. However, these may present particular challenges in southeast Asian contexts in which family reputation and maintaining family harmony are valued above individual liberty.^5^ Similarly, open discussion of sex and sexuality may be seen as cultural taboos, more so across generations and across genders.^5^ Rather than transporting and inserting PE programmes across different locales as a presumably culture-neutral intervention and expecting similar processes and outcomes to transpire, our findings suggest the importance of local stakeholder engagement and formative research to anticipate challenges, such as those due to gender dynamics, social hierarchies, and local tensions between groups of young people. This finding is corroborated by a recent global systematic review and meta-analysis of 60 studies of PE programmes for HIV prevention among young people and adults from key populations, which identified evidence for greater PE programme effectiveness in promoting behaviour change outcomes in LMICs than in high-income countries. The authors recommend PE interventions as particularly well-suited for LMICs due to their relatively low costs, and indicate the need for additional research to assess social-environmental and structural factors that may impact on PE programme implementation and effectiveness.^17^

To that end, our findings suggest possible sociocultural and structural differences between countries within the Mekong Region that may impact on the effectiveness of PE programmes. Potentially influential axes of difference emerged in the degree of social hierarchy, characteristics of intergenerational relations, and the impact of gendered power norms in different geopolitical contexts. For example, ongoing individual and familial effects of intergenerational trauma due to the Cambodian genocide^54^ were described as reinforcing hierarchical generational strictures and adults’ felt need to safeguard young people, which created barriers to youth empowerment and adults’ relinquishing control over peer initiatives.^32^ Although social hierarchy and the primacy of family exerts a strong influence across the Mekong Region,^54^ there appeared to be greater flexibility in programmes in some other countries in the region that endeavoured to inculcate peer leadership, such as described among adolescents in Thailand.^34,39^

Notably, most of the studies reviewed did not address gendered differences outside a few one-off examples among cisgender youth. Cisgender young women were deemed more challenging to recruit among young people who use drugs in Vietnam^45^ and more constrained by gender norms in participating in mixed gender groups in Cambodia,^32^ with separate groups by gender in Thailand facilitating discussion among cisgender women.^48^ Although even less of a focus, cultural gender norms also emerged among sexual and gender minority youth. For example, in Vietnam, YMSM who were more visibly identifiable and those who self-identified as “straight” were deemed less willing to engage in PE interactions in public settings than other YMSM.^46^ Intersectional culture and gender dynamics suggest the importance of formative research conducted in local partnership with young people, including sexual and gender minorities, in intended PE programme locales, and the value of research strategies that engage with gender norms, and gender and sexual diversity. Future research on PE interventions for HIV prevention and sexual health with young people should provide gender- and sexual orientation-disaggregated data to evaluate programme effectiveness and inform subsequent intervention development.^4,6^ PE intervention research should also focus on under-researched countries (i.e. Cambodia, Lao PDR) and populations (i.e. transgender young people) in the Mekong Region.

**Figure 2.**
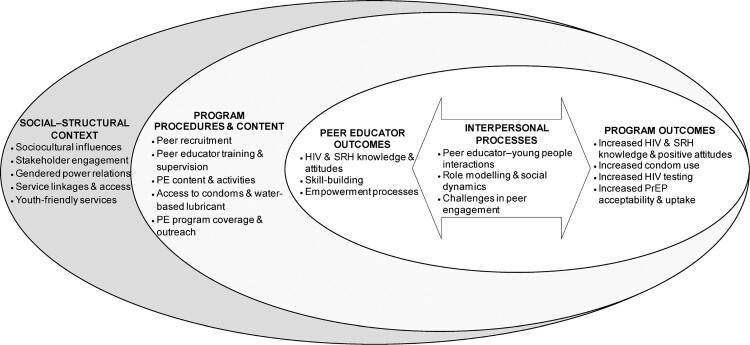
Social-structural and programme contexts, interpersonal processes, and peer educator and programme outcomes of peer education interventions for HIV prevention and sexual health: a conceptual model

Finally, based on a synthesis of empirical findings from this review, we developed a conceptual model (see Figure 2) informed by a rights-based approach to sexual health and HIV prevention.^12,18^ The model depicts PE interventions as inherently relational, foregrounding interpersonal processes between peer educators and young people, and positions peer educator outcomes as complementary to programme outcomes for target populations of young people. PE interventions, in turn, are subsumed within programmematic, and sociocultural and structural contexts,^18^ on which the implementation and effectiveness of PE interventions are contingent.^14,19^ This is in contrast with approaches that elide peer educator outcomes as outside the scope of PE programme effectiveness and that underemphasise broader sociocultural factors, such as gender norms, sexual and gender diversity, and youth empowerment, which are central to rights-based approaches.^12^ Collectively, this model suggests a shift in research and implementation of PE interventions for young people, from a laser focus on assessment of fixed behavioural and biological outcomes conceptualised as external to PE interventions, to one that encompasses and assesses dialectical and relational processes, and the situated implementation of PE interventions in complex sociocultural systems – an evidence-making intervention approach.^6,55^ Evidence-making approaches may be particularly valuable in engaging with marginalised populations at elevated risk for HIV infection, such as sexually and gender diverse young people, and in sociocultural and political contexts that constrain young people’s engagement, decision-making, and the broader implementation of programmes to support HIV prevention, and sexual health and rights.^6^

In addition to the strengths of this review, limitations may arise from the exclusion of studies that were not written in English or indexed in major scholarly databases; however, we reviewed thousands of potential sources to derive the included studies and used rigorous methods to search, identify, and extract information. As this is a scoping review, rather than a meta-analysis, further research is needed to assess PE programme effectiveness and correlates of effectiveness; and due to the very few single-component PE interventions identified in our review, we are unable to make meaningful comparisons with multicomponent interventions. Nevertheless, much of the research on PE interventions for HIV prevention has tended to focus on immediate outcomes in young people’s knowledge and behaviours to the exclusion of other important programme dimensions,^13,21^ which emerged as integral in this review. To that end, qualitative and mixed methods studies are needed to illuminate cultural influences and social processes in PE interventions, and impacts beyond young people’s sexual health and HIV risk.^16,21,50^ Lastly, although we remained cognisant of representing diversity in the data presented, the paucity of studies from Lao PDR and Cambodia limited our ability to corroborate findings within these countries.

## Conclusions

This review suggests the need to expand investigations of PE interventions for promoting HIV prevention and sexual health among young people through increased focus on young key populations at risk for HIV, elements of PE programme implementation, and conceptualisations of PE programme effectiveness. An enhanced focus on evaluating the salutary impacts of PE processes among peer educators^49^ can help to advance multifocal considerations of the benefits of PE interventions; some of these may support longer-term outcomes and health-promoting shifts in local ecologies, which are typically outside the scope of most studies and evaluations.^25^ Future research should also aim to address knowledge gaps regarding location-population-specific sociocultural influences on PE programmes in order to anticipate challenges and opportunities for developing or adapting PE interventions in new contexts.^56^ Overall, these findings suggest the benefits of complementing traditional evidence-based approaches singularly focused on pre-defined and often externally mandated PE intervention methods and outcomes with that of evidence-making processes.^55^ An epistemic shift to an evidence-making approach is particularly suited to advancing the design, implementation, and evaluation of rights-based and rights-focused PE interventions,^12^ in which young people, their social interactions, and sociocultural milieu constitute focal processes and outcomes of PE interventions.

## Supplementary Material

Supplemental file 1. Studies on peer education (PE) for HIV prevention or sexual health with young people in Mekong Region countries.
