# Supplementary material for: Peer education interventions for HIV prevention and sexual health with young people in Mekong Region countries: a scoping review and conceptual framework
Source: Sex Reprod Health Matters. 2022 Oct 28;30(1):2129374. doi: 10.1080/26410397.2022.2129374 (PMC9621210; doi:10.1080/26410397.2022.2129374)
Supplement: Supplemental file 1. Studies on peer education (PE) for HIV prevention or sexual health with young people in Mekong Region countries. [file ZRHM_A_2129374_SM9840.docx]

**Supplemental file 1. Studies on peer education (PE) for HIV prevention or sexual health with young people in Mekong Region countries**

| **Study** | **Setting** | **Pop.** | **Sample size (n)** | **Age range (mean), years** | **Objective(s)** | **Methods** | **Program description** | **Main findings** |
| --- | --- | --- | --- | --- | --- | --- | --- | --- |
| Aung et al. (2014) | Myanmar, 7 cities: Yangon, Mandalay, Myitkyina, Monywa, Tachileik, Pyay, Bago | FSWs | 978 | 18 – 49 (23) | - To measure coverage of HIV prevention programs among young FSWs | Quant  (cross- sectional surveys) | Survey of exposure to components of HIV prevention programs, including PE, & behavioral risk indicators | - 15% – 50% (median, NR) attended a peer educator talk - 8% – 67% (median 31%) visited a drop-in center (past 3 mos.) - 8% – 73% (median 43%) HIV tested & received results (past yr.) - 88% – 99% consistent condom use with clients (past wk.) - all prevention approaches need scale-up in Yangon |
| Aung et al. (2017) | Myanmar, 10 townships | YMSM | 1,657 | 15 – 24 (20) | - To evaluate effectiveness and acceptability of Link Up intervention | Mixed:  (quasi-experimental) | Multi-component, community- and clinic-based   - PE & outreach (free condoms & lubricant, HIV/STI prevention counselling, referrals) - Peer educator training (individual & group education on health promotion, HIV/ STIs, gender/sexuality, & gender-based violence) - Clinic staff training on sexual health & rights of YMSM/youth-friendly services - HIV/STI testing - Integrate community & clinic services | ↑ access HIV testing services (NS)  ↑ HIV-related knowledge (NS)   - perceived as acceptable (qual results) |
| Broadhead et al. (2009) | Vietnam: Ha Giang (China, Guigang) | IDUs | 610 Vietnam (total, 892) | < 25  56% of sample | - To investigate feasibility of recruitment and education through a PDI in the cultural context | Quant  (quasi- experimental /pre-/post-test) | Multi-component, community-based   - PE & outreach - Health educator training - Referrals to services - Distribute HIV prevention leaflets - Condom distribution - Needle/syringe exchange | ↓ lending, borrowing or sharing syringes  ↑ willingness to enter drug treatment   - suggested effectiveness of IDU recruiters in educating and recruiting for intervention services, supporting high feasibility |
| Chamratri-thirong et al. (2017) | Thailand, 28 factories | Young factory workers | 699 | 18 – 24 (21) | - To investigate effects of a workplace program intervention on AIDS knowledge, perceived access to condoms, and condom use | Quant  (cross-sectional survey) | Multi-component, workplace-based   - Policy development for management of HIV+ employees - HIV/AIDS training sessions for managers, peer leaders, workers - Distributed educational materials - Condom distribution | ↑ AIDS knowledge  ↑ perceived access to condoms  ↑ condom use with regular partners |
| Fongkaew et al. (2007) | Thailand, 10 schools in Chiang Mai province | Adolescents & young adults | 2,147 | Youth leader trainers, 16 – 20  Younger youth leaders, 10 – 14 | - To develop & evaluate HIV prevention program using participatory action research (PAR) | Mixed:  (PAR, quasi- experimental and focus group) | Multi-component, school-based   - Partnership: researchers & 10 schools - Researcher-teacher workshops - Curriculum development - Enhance capacity of youth volunteers to become youth leader trainers (YLTs) - Skill-building camps for younger youth leaders (YYLs), including parents - Youth-adult partnership using participatory learning experiences - Peer-led activities in schools, ‘edutainment’ | ↑ SRH knowledge  ↑ positive attitudes towards sexual health   - peer educators developed confidence in leadership and enhanced communication skills |
| Fongkaew et al. (2011) | Thailand, 12 schools from 3 districts in Chiang Mai | Adolescents | 2,492 | Senior youth leaders, 16 – 18;  Jr youth leaders, 11 – 12;  Target peers, 9 – 12 | - To develop & evaluate senior-junior peer program for SRH education & HIV prevention, using youth-adult partnership with schools approach | Mixed:  (PAR) | Multi-component, school-based   - Partnered research team & schools - Researcher-teacher workshops - Peer-led activities: leadership capacity, SRH education, HIV prevention - Role modelling - Building capacity of teachers - Public forums & innovative media - Policy recommendations | ↑ HIV/AIDS knowledge  ↑ positive attitudes towards sexual health   - integration of HIV prevention activities in the school system |
| Khoat et al. (2003) | Vietnam, 61 provinces and urban areas | IDUs, CSWs, PLHIV | 980 | IDUs (NR)  CSWs 16 – 19 (> 50%)  PLHIV (NR) | - To assess number & coverage of PE programs for HIV prevention | Mixed:  (program evaluation) | Multi-component, community-based   - PE & outreach - Condom, syringe & needle distribution - Peer educator training - Home-based care for PLHIV - Educational materials - Referrals for HIV testing & rehab | - 20 (32%) of Vietnam’s 61 provinces & urban areas had a functioning PE program - coverage limited in some provinces despite high number of PLHIV - most provinces reported concerns about program funding & sustainability |
| Knibbs and Price (2009) | Cambodia, urban, peri-urban & rural community sites | Young people | 208 | 18 – 25 | - To analyze inter-relationships between political & social factors, & PE interventions - To explore impact & effectiveness of PE to address SRH needs | Qual  (case study) | Multi-component, community-based   - - PE & outreach   - Condom distribution   - Peer educator training   - Group sessions & project activities | - PE tenets of empowerment & participation conflicted with specific hierarchical traditions & local power relations: gender & poverty - Peer educators trained to deliver messages developed by adults - Interventions not designed to reflect youth peer group dynamics |
| Longfield et al. (2011) | Lao PDR, 3 cities: Luang Prabang, Vientiane, Savan-nakhet | Male-to-female, *kathoey*, TGW | 703 | 15 – 35, (22, in 2004; 21, in 2006) | - To identify changes in key behaviors & correlates of HIV risk, & association with exposure to *kathoey*-specific social marketing intervention | Quant  (cross-sectional survey) | Multi-component, community-based   - PE & outreach - Peer educator training & workshops - Peer-led interpersonal communication - “Friendly” drop-in centers - Camping trip, health education & life skills trainings | ↑ condom use with casual partners (last anal sex)  ↑ water-based lubricant use  ↑ condom use knowledge   - Intervention was deemed feasible |
| Mimiaga et al. (2016) | Vietnam, Ho Chi Minh City | MSM | 100 | 18 – 25 (81% of sample) | - To determine feasibility & acceptability of Men’s Small Group intervention among a mixed HIV serostatus sample | Mixed:  (pilot study) | Multi-component, community-based   - PE & outreach - Week-long in-depth training of peer-facilitators - Group-based sexual risk reduction sessions & activities | ↓ condomless anal sex at 3-mos. & 6-mo. follow-up   - Intervention feasibility & acceptability evidenced by 87% retention, 78% completion of 6-mo. assessment, positive evaluations, & qual. exit interviews |
| Ngo et al. (2013) | Vietnam, 5 provinces | Young people | 1,212 | 15 – 24 | - To investigate changes in behavioral outcomes related to PE & integration of HIV voluntary counseling and testing (VCT) & SRH services | Quant (pre-test/post-test, non-experimental evaluation) | Multi-component, clinic and community-based   - PE & outreach - Peer educator trainings - Trained clinic medical staff, counselors & lab technicians (youth-friendly services) - Distribute information, education & communication (IEC) materials - Integration of VCT into SRH services - Referral systems to VCT & SRH - Link communities to free VCT services | ↑ HIV testing intentions, 33% to 51%  ↑ ever tested, 7.5% to 15%  ↑ repeat tested (past 12 mos.)  ↑ 5-fold seeking VCT in government SRH clinic sample, 5% to 25%  ↑ 2-fold last HIV tested at a project-supported clinic, 9% to 18% |
| Oldenburg et al. (2014) | Vietnam, Ho Chi Minh City | MSWs | 281 | 15 – 48 (21) | - To assess factors associated with access to PE & peer educators contact, & willingness to take PrEP | Quant  (survey) | Survey   - HIV testing knowledge - HIV/STI testing history - Contact with PE, previous 12 mos. - Willingness to take PrEP | Contact with peer educators: 68%  ↑ 2-fold willingness to use PrEP  ↑ peer educators contact assoc. w/older age  ↓ peer educators contact assoc. w/ ‘effeminate’ or ‘straight boy’ vs. ‘masculine’   - 95% willing to use PrEP - 57% willing to use PrEP with side effects |
| Ongwan-dee et al. (2018) | Thailand, 5 hospitals in 4 provinces | MSM, TGW | 1,880 | 20 – 29 (23) | - To increase HIV testing coverage - To identify barriers to immediate initiation of ART & PrEP | Quant  (cross- sectional survey) | Multi-component hospital-/clinic-based   - - PDI to expand recruitment   - Peer educator & healthcare worker training (stigma & discrimination, benefits/risks of HIV testing)   - PrEP information sheets   - HIV counseling & rapid testing   - HIV treatment & PrEP services - One-stop services (hospital/clinic) | - 70% (n=1,312) tested for first time - 16% HIV+ - 31% of HIV-‘s started PrEP   Peer vs. clinic recruited:  ↓ age  ↓ HIV-infected  ↑ CD4 count among PLHIV   - compensated peer-driven recruitment can reach MSM & TGW at high risk of HIV infection |
| Sherman et al. (2009) | Thailand, Chiang Mai province | Young meth-users | 983 | 18 – 25 (19) | - To compare efficacy of a PE network-oriented intervention vs. best practice life-skills curriculum on methamphetamine use, sexual behaviors, and incident STIs | Quant  (RCT) | Multi-component, community- & hospital-based   - - PE to reduce methamphetamine use & sexual risk behaviors   - Peer educator & life skills training   - HIV pre- and post-test counselling - Referrals for medical treatment | Both programs:  ↓ self-reported methamphetamine use (99% baseline vs. 53% 12 mos.)  ↑ consistent condom use (32% vs. 44%)  ↓ incident STIs (12 mos.)   - no significant difference between PE vs. life-skills study arms |
| Thato and Penrose (2013) | Thailand, university in Bangkok | Young adults,  college students | 505 | Peer leaders: 19 – 22 (20)  Peers: 18 – 22 (20) | - To test the effectiveness of A Brief, Peer-Led, HIV Prevention Program (BPL-HIV) | Quant  (quasi-experimental research/) | Multi-component, school-based   - - PE & outreach   - Educational & entertaining activities - Peer leaders received healthy sexuality course, HIV prevention activity protocol, & visited/interviewed PLHIV | ↑ HIV prevention knowledge  ↑ attitudes to preventive behaviors  ↑ HIV prevention subjective norms  ↑ condom use intentions & safer sex discussion intentions   - No difference in preventive behaviors among sample (10%) reporting sexual intercourse (past 6 mos.) |
| Veronese et al. (2018) | Myanmar, 2 cities; Yangon & Mandalay | MSM, TGW | 425 | 20 – 28 (25) | - To investigate the acceptability of peer-delivered HIV testing among MSM & TGW from existing HIV prevention programs | Quant  (cross sectional surveys) | Survey of clients of multi-component, community-based program   - - Peer-delivered HIV testing education & counselling   - Distribution of condoms & lubricants   - Outreach activities   - Drop-in centers - Peer educators trained to recruit participants | Peer-delivered HIV testing acceptability associated with:  ↑ *Apone* (‘masculine’/gay MSM) vs. *Apwint* (TGW) or *Tha Nge* (heterosexually-identified MSM*)*  ↑ perceived HIV risk  ↑ ≥ 5 casual partners (past 3 mos.)   - 86% ‘comfortable/very comfortable’ with peer-delivered testing |
| Wasantioo-papokakorn et al. (2018) | Thailand, 4 public hospitals | MSM, TGW | 5,609 | 18 – 31 (24) | - To increase HIV testing & linkage to care for HIV+ through peer-led counseling & referrals, training of hospital staff, & mobile HIV testing | Quant  (longitudinal cohort study) | Multi-component hospital-/clinic- and community-based   - Peer educators trained in sampling & recruitment, providing HIV education & HIV testing referrals - Sensitivity training for hospital staff - Rapid HIV testing - Referrals to care and treatment | - 21% (n=1,193) tested HIV+   ↑ HIV testing from 2012 (n=458) to 2016 (n=1,832)   - mobile clinic- vs. hospital-tested   ↓ age  ↑ referred by peer educator  ↑ first time HIV tested  ↓ HIV-infected  ↑ CD4 count   - highest HIV incidence among 20 –24 y.o. (10.9 per 100 person-years) |

ART, antiretroviral therapy; CSW, commercial sex worker; FSW, female sex worker; IDU, injection drug user; Mixed, mixed methods; MSM, men who have sex with men; MSW, male sex worker; NR, not reported; NS, not significant; PAR, participatory action research; PDI, peer-driven intervention; PE, peer education; PLHIV, people living with HIV; Pop, populations; PrEP, pre-exposure prophylaxis; Qual, qualitative; Quant, quantitative; SRH, sexual and reproductive health; STIs, sexually transmitted infections; TGW, transgender women; VCT, voluntary counseling and testing; YMSM, young men who have sex with men
